# Cytotoxicity Produced by Silicate Nanoplatelets: Study of Cell Death Mechanisms

**DOI:** 10.3390/toxins12100623

**Published:** 2020-09-29

**Authors:** Jie-Ting Huang, Ling-Chu Chang, Chung-Ssu Cheng, Jiang-Jen Lin, San-Yuan Huang, Shuen-Ei Chen

**Affiliations:** 1 Department of Animal Science, National Chung Hsing University, Taichung 40227, Taiwan; jieting2283@gmail.com (J.-T.H.); terry80149@hotmail.com (C.-S.C.); syhuang@dragon.nchu.edu.tw (S.-Y.H.); 2 Chinese Medicinal Research and Development Center, China Medical University Hospital, Taichung 40447, Taiwan; t27602@mail.cmuh.org.tw; 3 Center for Molecular Medicine, China Medical University Hospital, Taichung 40447, Taiwan; 4 Department of Biological Science and Technology, China Medical University, Taichung 40402, Taiwan; 5 Department of Materials Science and Engineering, National Chung Hsing University, Taichung 40227, Taiwan; jianglin@ntu.edu.tw; 6 The iEGG and Animal Biotechnology Center, National Chung Hsing University, Taichung 40227, Taiwan; 7 Innovation and Development Center of Sustainable Agriculture (IDCSA), National Chung Hsing University, Taichung 40227, Taiwan; 8 Research Center for Sustainable Energy and Nanotechnology, National Chung Hsing University, Taichung 40227, Taiwan

**Keywords:** nano-silicate platelets, necroptosis, reactive oxygen species, endocytosis, membrane integrity

## Abstract

Nano-silicate platelets (NSP), an exfoliated product from natural clays, have been validated for biosafety and as an effective supplement to alleviate mycotoxicosis. Since NSP induced noticeable cell death, we therefore investigated further the mechanism of cytotoxicity caused by NSP. Exposure to NSP impaired membrane integrity and caused cell death in a dose-dependent manner. Reactive oxygen species (ROS) generation other than of NADH oxidase origin, and subcellular interactions by internalized NSP also contributed to NSP-induced cell death. NSP persistently provoked receptor-interacting protein 1 Ser/Thr (RIP1) kinase and caspase 6 and 3/7 activation without altering caspase 8 activity and induced evident chromatolysis of necrosis in the later stage. These events proceeded along with increased ER stress and mitochondrial permeability, to final Cyt-C (Cytochrome C) release and AIF (apoptosis inducing factor) translocation, a hallmark of cell necroptosis. Fluorescent probing further manifested NSP traffic, mostly adherence on the cell surfaces, or via internalization, being compartmentalized in the nuclei, cytosols, and mitochondria. Pharmacological approaches with specific inhibitors suggested that endocytosis and particularly RIP1 kinase provocation mediate NSP-induced cell death independent of caspase activation. In conclusion, the necroptotic process contributes to most of the cell death induced by NSP due to membrane interactions/impaired integrity, ROS generation, and subcellular interactions by internalized NSP.

## 1. Introduction

Conventionally, sheet silicate minerals from phyllosilicates, including bentonite and montmorillonite clays, have been used as medical materials for diarrhea in humans, due to their antibacterial activity [1]. In livestock management, these aluminosilicate clays, as well as purified sodium calcium aluminosilicates (HSCAS), have been widely used as a supplement to alleviate mycotoxicosis in poultry and swine [2].

Aluminosilicate-containing clays exhibit a strong interaction attracting polar functional groups of mycotoxins depending on pore size, charge intensity, and intrinsic affinity and thereby hinder the absorption of mycotoxins in the digestive tract [3,4]. The core aluminum ions of HSCAS can interact with the β-carbonyl group of aflatoxins (AFB1) leading to a high affinity to AFB1 [5]. Despite being quite effective in detoxifying aflatoxicosis, HSCAS products are less effective in counteracting toxicosis by *Fusarium* mycotoxins, such as fumonisins, trichothecenes, and zearalenone [2].

Previously, we demonstrated that exfoliated sheet-like aluminosilicate clays serve as an effective feed supplement to alleviate marker pathologies and improve growth performance in chickens intoxicated with fumonisin B1 (FB1) or AFB1 [6,7]. As low as 40–200 mg/kg feed of exfoliated silicate nanoplatelets were able to counteract toxicosis by FB1 and AFB1. Dietary inclusion of nano-silicate platelets (NSP) also promoted feed intake and growth in chickens as well as the alleviation of FB1 toxicosis [6]. In pregnant mice intoxicated with FB1, oral administration of NSP at 2.5 mg/kg BW suppressed maternal plasma FB1 concentrations by 93%, ameliorated embryo neural tube defects and teratogenesis, and thus significantly improved fetus growth [8].

The exfoliative process with aluminosilicate clays including montmorillonite and bentonite afforded NSP nano-clays that are finely dispersible in water and possess physical properties of a polygonal geometric shape of one nanometer thickness (ca. 80 × 80 × 1 dimension) with high surface area (ca. 720 m^2^/g), cationic exchange capacity at 1.20 meq/g, and intensive ionic charges (ca. 20,000 ions/platelet) [9,10,11]. These unique characteristics endow NSP with a high affinity to adhere to the surface of bacteria, and thereby with a strong bacteriostatic and bactericidal activity [11,12,13,14,15]. Activation of death signaling and reactive oxygen species (ROS) provocation irrelevant to Ag^+^ cytotoxicity was further shown to mediate the bactericidal effects of AgNP (silver nanoparticle)/NSP nanohybrids on silver-resistant *E. coli* [13,14,16]. Surfactants were also used to modify NSP at noncytotoxic concentrations and provoke a broad and potent antiviral activity due to electrostatic interactions with the virus in order to block the viral access to cell surfaces [17]. In in vivo studies, sodium dodecyl sulfate (SDS) surfactant facilitated NSP in shielding off infection by dengue, Japanese encephalitis, and influenza A virus, resulting in a reduction of lethality in the infected mice [17].

For practical applications, NSP was evaluated for biosafety and showed a very low toxicity, the lethal dose (LD50) being >5700 mg/kg of body weight in an acute toxicity study, in which rats receiving daily oral administration of NSP for 2 weeks exhibited normal livability, body weight change, feed intake, behaviors, and histology [12]. Despite the absence of genotoxicity, a very low but noticeable induction of cell death was observed when cells were exposed to NSP at levels > 62.5 μg/mL [12]. For further understanding of the safety issues, we embarked on a thorough investigation of the cytotoxicity and the mechanistic aspects of cell death by NSP induction.

## 2. Results

### 2.1. Cell Death by NSP Exposure

**Figure 1:**
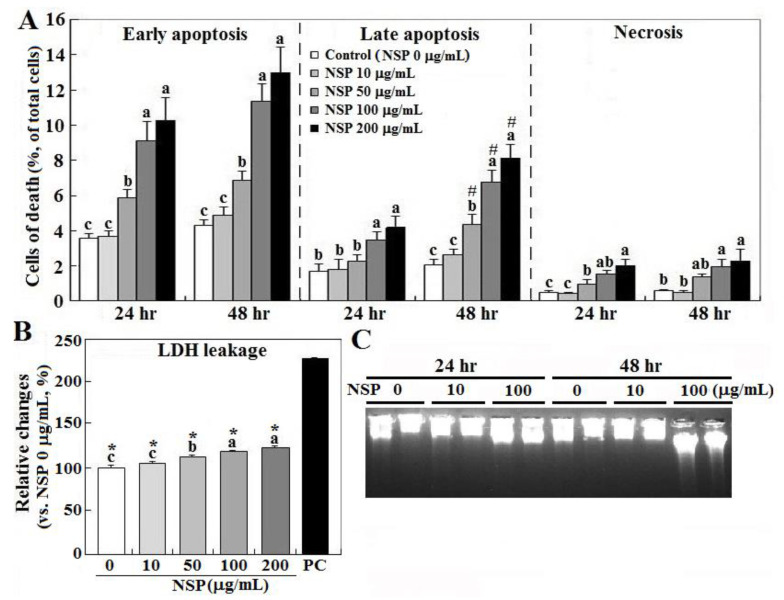
Effects of nano-silicate platelets (NSP) on cell death, lactate dehydrogenase (LDH) leakage, and DNA breakdown. Cells incubated with various levels of NSP for 24 or 48 h were collected for cell death (panel **A**), LDH leakage (24 h, panel **B**), and DNA breakdown analysis (panel **C**) (n = 4). Means with different superscript letters differ significantly among various levels of NSP treatment (*p* < 0.05). #; significant difference vs. 24 h treatment within the same type of cell death (*p* < 0.05). *; significant difference vs. positive control in LDH leakage (*p* < 0.05). Medium and cell lysates from the cultures without NSP supplementation (0 μg/mL) serve as the negative and positive (PC) control, respectively.

Exposure to NSP induced cell death in a dose-dependent manner, in which early apoptosis accounted for most cell death (10.1 and 13.1% at 24 and 48 h by 200 μg/mL, respectively), and interestingly only late apoptosis increased during the time course (3.7 to 7.8%), whereas necrosis contributed to cell death less than 2% (*p* < 0.05, Figure 1, panel A). NSP treatment for 24 h also increased cellular LDH (lactate dehydrogenase) leakage in a dose-dependent manner, suggesting damaged membrane integrity (*p* < 0.05, Figure 1, panel B). Instead of typical laddering fragmentation of DNA breakdown in apoptosis, cell death proceeded to necrotic chromatinolysis with a more pronounced smearing pattern of DNA size in electrophoresis after exposure to NSP for 48 h (Figure 1, panel C).

### 2.2. ROS Contribution and Origins

**Figure 2:**
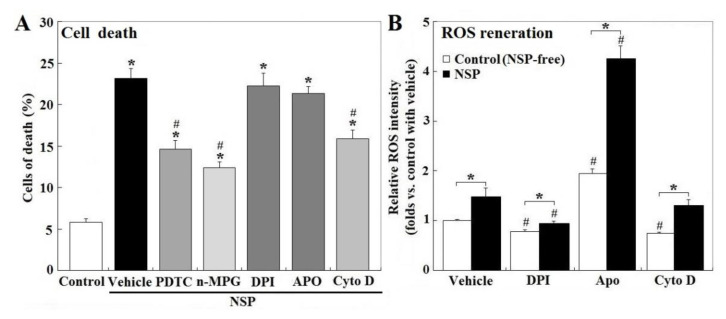
Effects of reactive oxygen species (ROS) scavenging and blockade of NADH oxidase and endocytosis on NSP-induced cell death and ROS production. Cells pre-treated with vehicle or pharmacological inhibitors were incubated with 100 µg/mL NSP for 48 h and then harvested for cell death analysis (panel **A**) or for 3 h for ROS generation analysis (panel **B**) (n = 4). Results of cell death were combined with early, late apoptosis and necrosis. *; significant difference by NSP treatment (vs. control, *p* < 0.05). #; significant difference by inhibitor treatment (vs. corresponding group in vehicle treatment, *p* < 0.05). PTDC; ammonium pyrrolidinedithiocarbamate, n-MPG; n2-mercaptopropionyl-glycine, Cyto D; cytochalasin D, DPI; diphenyleneiodonium chloride, Apo; apocynin, NADH; Nicotinamide adenine dinucleotide.

Treatment of PTDC (ammonium pyrrolidinedithiocarbamate) and n-MPG (n2-mercaptopropionyl-glycine) for ROS scavenging, or Cyto D (cytochalasin D) to block endocytosis, but not by NADH (nicotinamide adenine dinucleotide) oxidase inhibitors, DPI (diphenyleneiodonium chloride) or Apo (apocyni), partially rescued cell death induced by NSP (*p* < 0.05, Figure 2, panel A). Exposure to NSP induced ROS production regardless of the presence of the pharmacological inhibitors (*p* < 0.05, Figure 2, panel B). In contrast to their respective vehicle control, ROS production was suppressed by DPI but increased by Apo in both vehicle control and NSP-treated cells (*p* < 0.05, Figure 2, panel B), consistently with previous studies which showed that Apo represses NADH oxidase activity only in phagocytic cells, but stimulates ROS generation in non-phagocytic cells [18]. In contrast to the vehicle control, Cyto D treatment suppressed ROS generation in NSP-free conditions (*p* < 0.05, Figure 2, panel B), but not in the presence of NSP. In combination with results from Figure 1, ROS generation other than by NADH oxidase origin, and intracellular interactions/mechanisms activated by internalized NSP irrelevant to ROS generation, were concluded to contribute to NSP-induced cell death.

### 2.3. RIP1 Kinase, Caspase Activation, and ER Stress

NSP exhibited a high affinity to adhere onto cell surfaces [12] and induced cell death with necrotic chromatinolysis rather than laddering fragmentation of DNA breakdown in apoptosis (Figure 1). Membrane integrity impairment/interactions, ROS provocation, and the endocytic process were shown to contribute to NSP-induced cell death (Figure 1 and Figure 2). We then studied the type of cell death and its progression.

**Figure 3:**
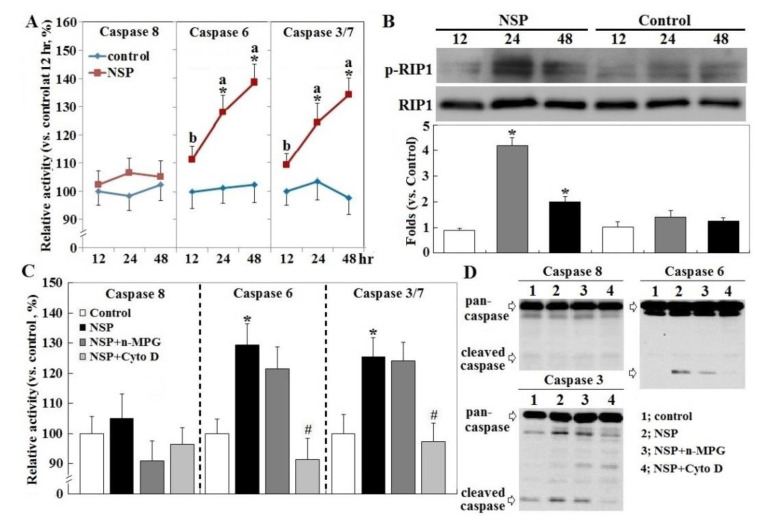
Receptor-interacting protein 1 Ser/Thr (RIP1) activation, and effect of ROS scavenging and endocytosis blockade on NSP-induced caspase activation. Cells alone (panel **A**), or pre-treated with vehicle or pharmacological inhibitors, were incubated with 100 µg/mL NSP for 12, 24 or 48 h and then harvested for caspase (panel **C** at 24 h, panel **D** at 48 h) and RIP activation (panel **B**) analysis through enzymatic or Western blot method (n = 4). Means with different superscript letters differ significantly among different time points (*p* < 0.05). *; significant difference by NSP treatment (vs. corresponding control, *p* < 0.05). #; significant difference by inhibitor treatment (vs. NSP treatment, *p* < 0.05). n-MPG; n2-mercaptopropionyl-glycine, Cyto D; cytochalasin.

**Figure 4:**
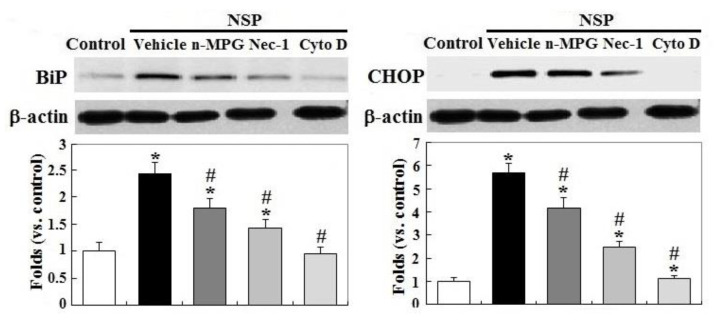
Effects of ROS scavenging and blockade of endocytosis and RIP1 kinase on SP-induced ER stress. Cells pre-treated with vehicle or pharmacological inhibitors were incubated with 100 µg/mL NSP for 24 h and then harvested for BiP and CHOP (C/EBP homologous protein) expression through Western blot analysis (n = 4). *; significant difference by NSP treatment (vs. control, *p* < 0.05). #; significant difference by inhibitor treatment (vs. vehicle with NSP treatment, *p* < 0.05). n-MPG; n2-mercaptopropionyl-glycine, Cyto D; cytochalasin D, Nec-1; necrostatin-1.

Exposure to NSP persistently promoted RIP1 (receptor-interacting protein 1 Ser/Thr) kinase activation and caspase 6 and 3/7 activity, two executioners in cell apoptosis, but not in caspase 8 (*p* < 0.05, Figure 3, panel A and B). The induction of caspase 6 and 3/7 activity by NSP was completely reversed by Cyto D but not by n-MPG, whereas neither n-MPG nor Cyto D affected caspase 8 activity (*p* < 0.05, Figure 3, panel C and D). Since caspase 8 mediates the extrinsic apoptotic signaling pathway and acts as a RIP1 kinase repressor, whose activity determines cell death by apoptotic or necroptotic process [19,20], these results thus exclude the activation of the extrinsic apoptotic pathway and suggest the necroptotic process and intrinsic apoptosis involved in the progression of NSP-induced cell death. It is concluded that ROS generation mediates NSP-induced cell death in a caspase-independent manner. Treatment of n-MPG and Nec-1 (Necrostatin-1, a RIP1kinase inhibitor) relieved ER (endoplasmic reticulum) stress by NSP as evidenced by downregulation of BiP and CHOP (C/EBP homologous protein) expression (*p* < 0.05, Figure 4). Blockade of endocytosis by Cyto D completely abolished the increases of ER stress caused by NSP (*p* < 0.05, Figure 4), suggesting that physical interactions with subcellular organelles or components such as proteins, and subsequent mechanisms provoked by internalized NSP, promote ER stress.

### 2.4. Mitochondrial Membrane Potential, Cyt-C Release, and AIF Translocation

**Figure 5:**
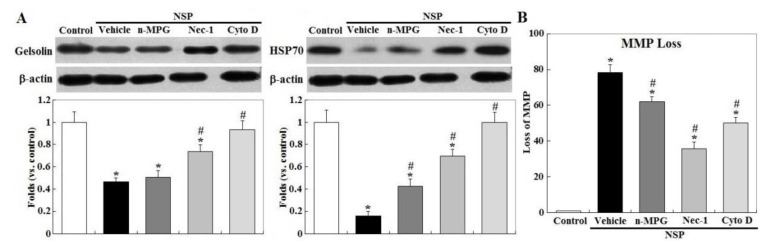
Effects of ROS scavenging and blockade of endocytosis and RIP1 kinase on NSP-induced expression of gelsolin, heat shock protein 70 (HSP70) and mitochondrion membrane potential. Cells pre-treated with vehicle or pharmacological inhibitors were incubated with 100 µg/mL NSP for 24 h and then harvested for gelsolin and HSP70 expression by Western blot (panel **A**) or for MMP (mitochondrial membrane potential) (panel **B**) analysis (n = 4). *; significant difference by NSP treatment (vs. control, *p* < 0.05). #; significant difference by inhibitor treatment (vs. vehicle with NSP treatment, *p* < 0.05). n-MPG; n2-mercaptopropionyl-glycine, Cyto D; cytochalasin D, Nec-1; necrostatin-1.

**Figure 6:**
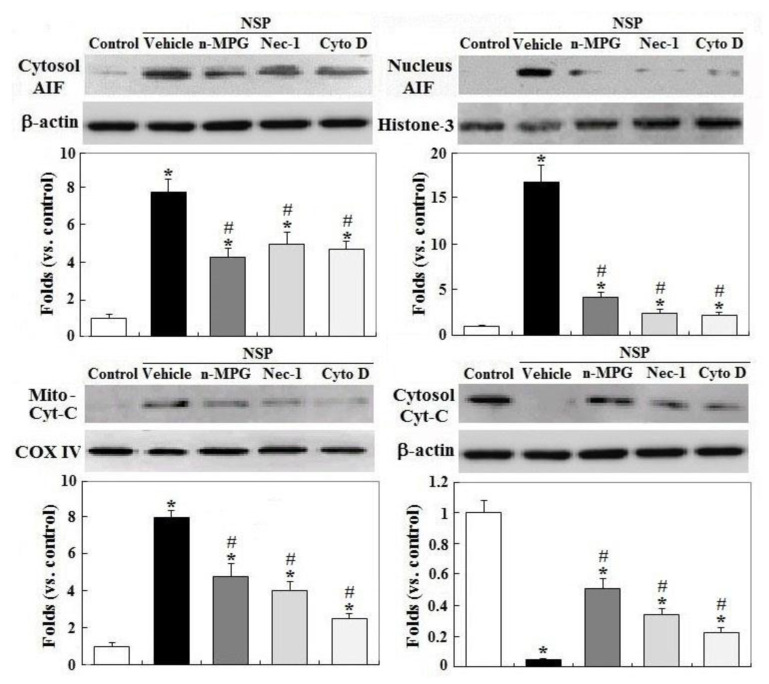
Effects of ROS scavenging and blockade of endocytosis and RIP1 kinase on NSP-induced translocation of AIF (apoptosis inducing factor) and Cyt-C (cytochrome C) release. Cells pre-treated with vehicle or pharmacological inhibitors were incubated with 100 µg/mL NSP for 48 h and then harvested for fractionation for AIF and Cyt-C translocation analysis through Western blot method (n = 4). *; significant difference by NSP treatment (vs. control, *p* < 0.05). #; significant difference by inhibitor treatment (vs. vehicle with NSP treatment, *p* < 0.05). n-MPG; n2-mercaptopropionyl-glycine, Cyto D; cytochalasin D, Nec-1; necrostatin-1.

Treatment with Nec-1 and Cyto D, but not by n-MPG, differentially rescued downregulation of gelsolin by NSP exposure, a mitochondrial permeability stabilizer (*p* < 0.05, Figure 5) [21]. Downregulation of HSP70 (heat shock protein 70) by NSP, a protein chaperone which repressively interacts with caspase 9 and cytosolic AIF (apoptosis inducing factor) [22], was completely reversed by Cyto D and ameliorated by Nec-1 and n-MPG (*p* < 0.05). Consistent with these results, n-MPG, Cyto D, and Nec-1 differentially ameliorated mitochondrial membrane potential (MMP) loss by NSP, i.e., by rescuing mitochondrial membrane permeability (*p* < 0.05, Figure 5) and attenuating the downstream events including mitochondrial Cyt-C release, a critical activator for caspase cascading in intrinsic apoptosis, and AIF translocation into the nuclei where it induces chromatolysis, a hallmark of cell necroptosis (*p* < 0.05, Figure 6) [23].

### 2.5. Localization of NSP Traffic

**Figure 7:**
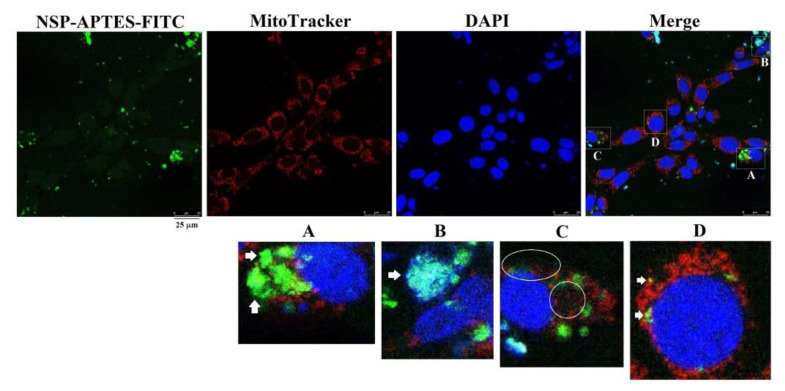
Subcellular compartmentalization of NSP. Cells were incubated with NSP-APTES-FITC (10 μg/mL) for 24 h. MitoTracker^®^ Red CMXRos was used to probe mitochondria. NSP-APTES-FITC were localized on the cell surfaces (panel **A**, bright green color), nuclei (panel **B**, cyan color), cytosol (panel **C**, scattered faint green color), and mitochondria (panel **D**, yellow color) as indicated by arrows or within circles. APTES; (3-aminopropyl) triethoxysilane, FITC; fluorescein isothiocyanate.

Morphological probing with fluorescent NSP-APTES-FITC localized NSP mainly aggregated to adherence onto the cell surfaces (Figure 7, panel A). Some NSP were ingested and trafficked to associate with the nuclei and mitochondria or scattered in the cytosols (panel B to D).

### 2.6. Caspase and RIP1/3 Kinase Activation, and NSP Internalization in NSP-Induced Cell Death

**Figure 8:**
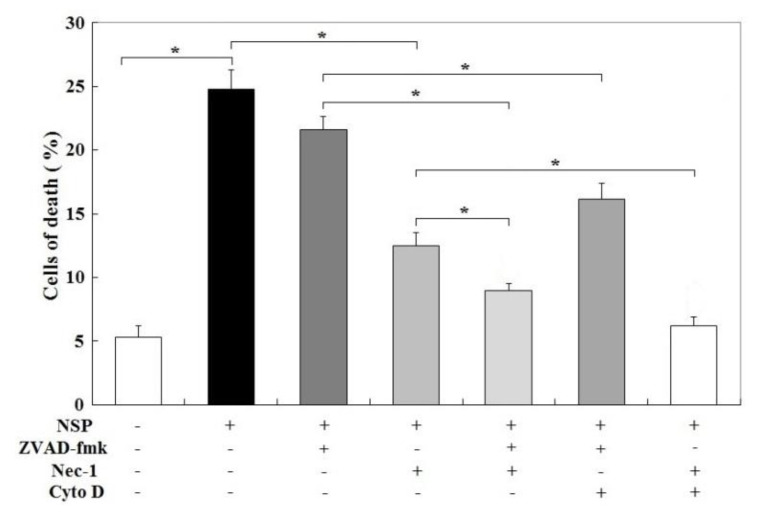
Effects of caspase, endocytosis, and RIP kinase blockade on NSP-induced cell death. Cells pre-treated with vehicle or pharmacological inhibitors were incubated with 100 µg/mL NSP for 48 h and then harvested for canalysis. Results of cell death were combined with early, late apoptosis and necrosis. *; significant difference (*p* < 0.05). Cyto D; cytochalasin D, Nec-1; necrostatin-1, ZVAD-fmk; Z-Val-Ala-Asp (OMe)-fluoromethyl ketone. +, −; present or absent with the indicated chemical(s).

Surprisingly, zVAD-fmk (Z-Val-Ala-Asp (OMe)-fluoromethyl ketone, a broad spectrum caspase inhibitor) failed, but Nec-1 partially rescued, NSP-induced cell death (*p* < 0.05, Figure 8). zVAD-fmk+Cyto D and zVAD-fmk+Nec-1 treatment also ameliorated NSP-induced cell death to a higher level than zVAD-fmk or Nec-1 treatment alone (*p* < 0.05), suggesting that necroptosis through RIP1/3 activation accounts for most NSP-induced cell death, whereas caspase activation and particularly internalized NSP synergistically participate in the programmed cell death. Treatment with Nec-1+Cyto D completely abolished cell death by NSP (*p* < 0.05).

## 3. Discussion

The present study defined cytotoxicity by NSP exposure including ROS induction, cell membrane interaction/damage, and intracellular interactions/mechanisms caused by internalized NSP in mediating cell death. This process of cell death evolved with RIP1 kinase activation in a caspase-independent manner, proceeded by downregulation of HSP70 and gelsolin, increased ER stress and mitochondrial permeability, and finally activated AIF translocation leading to cell necroptosis.

Most cell death by NSP exhibited apoptotic hallmarks including PS (phosphatidyl serine) exposure (annexin V-positive/Propidium iodide I-negative), mitochondrial Cyt-C release and caspase 6 and 3/7 activation. However, cells underwent death in the presence of zVAD-fmk and were sensitive to Nec-1, consistent with AIF translocation into the nuclei, necrotic smearing of DNA breakdown instead of laddering fragmentation, and no changes in caspase 8 activity.

Upon the activation of death receptors, caspase 8 repressively binds to RIP1 kinase and thus allows activation of apoptotic caspase signaling, but when caspase 8 is inhibited or activated inefficiently, RIP1 kinase can interact with RIP3 and drive cells into necroptosis [19]. Accordingly, these results suggest that the apoptotic pathway only accounts for a small part of NSP-induced cell death, which mostly occurs through necroptosis, a type of regulated cell death sharing some apoptotic phenotypes, but characterized by RIP1/3 kinase activation, AIF translocation into nuclei, and necrotic chromatolysis [19,20]. Apoptosis-like cell death, insensitive to zVAD-fmk but exacerbated by specific inhibition of caspase-8 activity, has been observed in NIH/3T3 cells in response to death receptor signaling [22] and in HepG2 cells induced by unmodified silicate clays [24].

Consistent with previous reports [11,12,13,14], a large portion of NSP was observed to adhere onto the cell membranes. This adherence may alter osmolality and polarization in local regions leading to impaired membrane integrity, provoke ROS generation, interact with local molecules such as death receptors, and thus activate death signaling [25]. Hyperpolarization of plasma membranes was shown to open calcium channels and resultant calcium cascades leading to ROS generation, calpain, and JNK (c-Jun N-terminal kinases) activation, which further act on mitochondrial PTPC (permeability transition pore complex) leading to AIF release into the cytosol, where AIF is activated by calpain processing [23,26].

In addition to functioning as a chaperone for proper protein folding and misfolded protein removal to relieve ER stress [27], HSP70 specifically represses AIF translocation and sequesters procaspase-9 from activation [23]. Gelsolin acts as a mitochondrial permeability stabilizer to prevent Cyt-C release [21]. Accordingly, downregulation of HSP70 and gelsolin by NSP may promote ER stress and facilitate AIF translocation and Cyt-C release to steer cells into the necroptotic process [28]. ROS provocation, other than by NADPH oxidase, mediates parts of NSP-induced cell death regardless of caspase activation, but is operative in mitochondrial permeability and AIF release. These results can be attributed to activation of necroptotic pathway, in which RIP1/3 kinase interacts with several mitochondrial metabolic enzymes and enhances autophagic degradation of catalase leading to ROS overproduction, and thereby impaired mitochondrial permeability [26].

Endocytosis blockade by Cyto D has been shown to ameliorate cytotoxicity involving ROS generation by amorphous nano-silica particles [29]. Non-phagocytic cells tend to ingest cationic nanoparticles depending on charge density and hydrophobicity [30]. As evidenced in the morphological probing study, high surface ionic charges of NSP promoted NSP ingestion into cells, and thus increased physical interactions with intracellular organelles leading to ROS generation and mitochondrial disruption, and subsequently cell death cascading. Physical interactions may also directly damage protein and DNA structure and translation machinery leading to ER stress, proteotoxicity and genotoxicity, when viewing the literature reporting the modification of nano-clays for susceptibility of ingestion by cells [30]. Ingestion of organically modified nano-clays has been shown to present in cytoplasmic vesicles and participate in cell death [31].

## 4. Conclusions

The present study concluded that ROS provocation, cell membrane interaction/damage, and intracellular interactions/mechanisms by internalized NSP mediate NSP-induced cell death. These cytotoxic processes operate by RIP1 kinase activation, ER stress and mitochondrial permeability, leading to AIF activation and ultimately cell necroptosis.

## 5. Materials and Methods

### 5.1. Preparations of NSP

Nanosilicate platelets were prepared from natural sodium montmorillonite (Na^+^-MMT) by exfoliation of the layered silicate clays using home-made polyamine-HCl as the exfoliating agent. Details of the synthetic procedures, purification, and characteristics of the NSP products were described previously [9,10,11].

### 5.2. Cell Cultures

NIH/3T3 mouse fibroblasts (ATCC, Manassas, VA, USA) were cultured in DMEM medium (Gibco, New York, NY, USA) supplemented with 10% fetal calf serum (Gibco) and 1% penicillin–streptomycin solution (Gibco), pH 7.4 at 37 °C, 5% CO2, 95% humidity. Medium was changed every 2 days. When reaching 80–90% confluence, cells were pre-treated with various pharmacological inhibitors for different durations including n-MPG (in distilled water, final concentrations 300 µM, Sigma-Aldrich, St. Louis, MN, USA), PDTC (in distilled water, 2 mM, Sigma-Aldrich), DPI (in DMSO, 10 µM, Calbiochem, San Diego, CA, USA), Apo (in DMSO, 30 µM, Calbiochem), and Cyto D (in DMSO, 2 nM, Sigma-Aldrich) for 30 min, zVAD-fmk (in DMSO, 50 µM, BioVision, Milpitas, CA, USA) for 1 h, and Nec-1 (in DMSO, 10 µM, BioVision) for 2 h. After replacing with new medium, cells were cultured with NSP and collected at indicated time points for further analyses. NSP were dispersed in PBS buffer with sonication for 10 min prior to treatment.

### 5.3. Cell Death and LDH Leakage Analysis

Cell death was analyzed by annexin-V/PI (propidium iodide) method using a commercial kit (BD Biosciences, San Jose, CA, USA) and flow cytometry for cell death sorting (FC500, Beckman Coulter Inc. Brea, CA, USA) within 1 h after staining [32,33]. Genomic DNA extracts were used for chromatinolysis analysis by evaluating DNA degradation under electrophoresis in 2% agarose gels. Cell lysates and culture medium were collected for LDH activity analysis using a commercial kit (Promega, Madison, WI, USA). Cell lysates and collected medium without NSP treatment were used as positive and negative control, respectively.

### 5.4. ROS Production, Mitochondrial Membrane Potential, and Caspase Activity

Cells pre-treated with various inhibitors or vehicles were cultured with 25 μM 2′,7′-dichlorofluorescin diacetate (DCFDA, Abcam, Cambridge, UK) at 37 °C in dark for 45 min. The cultures were then replaced with phenol red-free medium containing 100 µg/mL NSP and incubated for another 3 h. Generation of ROS was determined by fluorescence intensity with Ex/Em = 485/535 nm.

Determination of mitochondrial membrane potential (MMP, Δψm) was conducted as described previously [34] using 3,3′-dihexyloxacarbocyanine iodide [DiOC6(3), Molecular Probes Inc, Eugene, OR, USA] as a molecular dye. In brief, cells pre-treated with various inhibitors or vehicles were incubated with 100 µg/mL NSP for 48 h. Collected cells were suspended in PBS buffer containing DiOC6(3) (first dissolved in DMSO and diluted in PBS buffer, final concentration 40 nM). After incubation at 37 °C in dark for 30 min, cells were pelleted and washed with PBS to remove DiOC6(3). After 3 washes, MMP was measured by flow cytometric analysis (FC500, Beckman Coulter Inc.).

Caspase 6, 3/7, and 8 activities were determined as the cleavage rate of the synthetic fluorophoric (Biovision, Promega) or chromophoric (Invitrogen, Carlsbad, CA, USA) peptide substrate, respectively. Caspase 6 and 3/7 activity were read in with a microplate reader (Infinite F200 PRO, Tecan Group Ltd., Mannedorf, Switzerland) at Ex/Em = 400/505 and 480/520 nm, respectively, and caspase 8 activity was read at 400 nm.

### 5.5. Western Blot Analysis

Total cell lysates were prepared in RIPA (radioimmunoprecipitation assay) buffer containing protease and phosphatase inhibitor cocktail (Sigma-Aldrich). Isolation of cytoplasmic and nuclear fractions was conducted with the hypotonic buffer and centrifugation method as described previously [35]. Mitochondrial fractions were isolated according to the instructions enclosed in the commercial kit from Abacm. In electrophoresis for protein separation, each well contained a respective sample with 60 μg of proteins from total cell lysates, or 15–20 μg of proteins from cytoplasmic or nuclear fractions. Proteins were transferred onto PVDF (polyvinylidene fluoride) membrane with the wet-transfer method. A mouse anti-HSP70 (clone N27F3-4) monoclonal antibody was purchased from Enzo Life Sciences (New York, NY, USA). Rabbit anti-gelsolin (clone D9W8Y), anti-Cyt-C (clone 136F3), anti-COX IV (cytochrome c oxidase IV, clone 3E11), anti-caspase 8 (clone 1C12), anti-caspase 3 (clone D3R6Y), anti-RIP1 (clone D94C12) anti-phospho-RIP1 (clone D1L3S), anti-BiP (cone C50B12), anti-CHOP (clone D46F1), and anti-anti-β-actin (clone 13E5) monoclonal antibody were purchased from Cell Signaling Technologies (Danvers, MA, USA). A rabbit polyclonal anti-AIF, anti-histone 3, and anti-caspase 6 were derived from Aviva Systems Biology (San Diego, CA, USA), Abcam, and Cell Signaling Technologies, respectively. Horseradish peroxidase-conjugated secondary antibodies; goat anti-mouse IgG (Beckman Coulter, Brea, CA, USA) and anti-rabbit IgG (Calbiochem) were used for to identify the bands reactive to the primary antibodies through an enhanced chemiluminescence reagent (Pierce Biotechnology Inc., Rockford, IL, USA). Primary and secondary antibodies were incubated with membranes at 1:1000 and 1:7500–10,000 dilation, respectively.

### 5.6. Subcellular Compartmentalization of NSP

To visualize NSP traffics, NSP was tethered with biocompatible APTES dependents through alkaline catalysis by NH4OH (pH 11) to form NSP-APTES. Then, FITC was attached onto NSP-PHEMA to form NSP-APTES-FITC. Details of the preparations of NSP-APTES-FITC were described previously [36].

Cells grown on coverslips in a 6-well plate were cultured with NSP-APTES-FITC (10 μg/mL) for 24 h. Prior to cell collection, cells were incubated with MitoTracker^®^ Red CMXRos (Cell Signaling Technologies, Danvers, MA, USA) for 30 min and then fixed with 3.7% (*w/v*) paraformaldehyde and permeabilized with 0.2% (*v/v*) Triton X-100 for the determination of mitochondrial morphology. Coverslips were mounted and fluorescence images were taken by a Leica Microsystems TCS SP8 Confocal Spectral microscope (Leica Microsystems, Wetzlar, Germany) with Ex/Em = 579/599 nm for MitoTracker^®^ Red CMXRos and 493/528 nm for FITC.

### 5.7. Statistics

Data were analyzed by one-way ANOVA, in which NSP or pharmacological inhibitor treatments, or time point, were the classifying variables as indicated. Result were expressed as means ± SE. Differences between means were tested using Student’s *t*-test or Duncan’s multiple comparison procedure. All statistical procedures were performed by using SAS software (2000). Statistical significance was accepted at *p* < 0.05.
